# Mice with a Targeted Deletion of the Type 2 Deiodinase Are Insulin Resistant and Susceptible to Diet Induced Obesity

**DOI:** 10.1371/journal.pone.0020832

**Published:** 2011-06-16

**Authors:** Alessandro Marsili, Cristina Aguayo-Mazzucato, Ting Chen, Aditi Kumar, Mirra Chung, Elaine P. Lunsford, John W. Harney, Thuy Van-Tran, Elena Gianetti, Waile Ramadan, Cyril Chou, Susan Bonner-Weir, Philip Reed Larsen, Jorge Enrique Silva, Ann Marie Zavacki

**Affiliations:** 1 Thyroid Section, Division of Endocrinology, Diabetes and Hypertension, Brigham and Women's Hospital, Boston, Massachusetts, United States of America; 2 Section on Islet Transplantation and Cell Biology, Joslin Diabetes Center, Boston, Massachusetts, United States of America; 3 Longwood Small Animal Imaging Facility, Beth Israel Deaconess Medical Center, Boston, Massachusetts, United States of America; 4 Division of Endocrinology, Diabetes, and Metabolism, Baystate Medical Center, Springfield, Massachusetts, United States of America; University of Minnesota, United States of America

## Abstract

**Background:**

The type 2 iodothyronine deiodinase (D2) converts the pro-hormone thyroxine into T3 within target tissues. D2 is essential for a full thermogenic response of brown adipose tissue (BAT), and mice with a disrupted *Dio2* gene (D2KO) have an impaired response to cold. BAT is also activated by overfeeding.

**Methodology/Principal Findings:**

After 6-weeks of HFD feeding D2KO mice gained 5.6% more body weight and had 28% more adipose tissue. Oxygen consumption (V0_2_) was not different between genotypes, but D2KO mice had an increased respiratory exchange ratio (RER), suggesting preferential use of carbohydrates. Consistent with this, serum free fatty acids and β-hydroxybutyrate were lower in D2KO mice on a HFD, while hepatic triglycerides were increased and glycogen content decreased. Neither genotype showed glucose intolerance, but D2KO mice had significantly higher insulin levels during GTT independent of diet. Accordingly, during ITT testing D2KO mice had a significantly reduced glucose uptake, consistent with insulin resistance. Gene expression levels in liver, muscle, and brown and white adipose tissue showed no differences that could account for the increased weight gain in D2KO mice. However, D2KO mice have higher PEPCK mRNA in liver suggesting increased gluconeogenesis, which could also contribute to their apparent insulin resistance.

**Conclusions/Significance:**

We conclude that the loss of the *Dio2* gene has significant metabolic consequences. D2KO mice gain more weight on a HFD, suggesting a role for D2 in protection from diet-induced obesity. Further, D2KO mice appear to have a greater reliance on carbohydrates as a fuel source, and limited ability to mobilize and to burn fat. This results in increased fat storage in adipose tissue, hepatic steatosis, and depletion of liver glycogen in spite of increased gluconeogenesis. D2KO mice are also less responsive to insulin, independent of diet-induced obesity.

## Introduction

Thyroid hormone regulates a variety of processes including growth, development, and metabolic rate [1]. The thyroid gland produces predominantly thyroxine (T4), which has intrinsically low biological activity. However, this can be increased 10-times by the enzyme-catalyzed removal of an outer-ring iodine to produce 3,3′,5-triiodothyronine (T3) by the type 1 and 2 deiodinases (D1 and D2), an essential step in modulating thyroid hormone action. The type 3 deiodinase (D3), and under some conditions D1, can inactivate T3 and T4 by the elimination of an inner-ring iodine, generating 3,3′-T2 or reverse 3, 3′, 5′ T3 (rT3) respectively. Thus, the iodothyronine deiodinases modulate T3 action by regulating its production and degradation at both a systemic and tissue-specific level [2].

The D2 enzyme plays a key role in the local production of T3 from T4 within specific tissues. Over 30 years ago D2 activity was originally described when the mechanism of suppression of TSH in the pituitary by T4 was elucidated [3]. These findings were further confirmed in mice with a targeted deletion of the type 2 deiodinase gene (D2KO mice) where serum TSH levels were found to be decreased by treatment with T3, but not T4 [4]. The importance of D2-mediated local generation of T3 from T4 has also been established in many other diverse processes including chondrocyte differentiation, cochlear development, optimal bone strength and mineralization, and muscle regeneration after wounding [5]–[8].

Perhaps one of the best characterized functions of D2 is its essential role in mediating a full thermogenic response of brown adipose tissue (BAT) to adrenergic stimulation *via* increased T4 to T3 conversion within this tissue [9]–[11]. Further elegant studies in D2KO mice have shown that these animals are deficient in both lipolysis and lipogenesis leading to BAT dysfunction, and only survive cold exposure by a compensatory increase in shivering [12], [13].

BAT is considered a tissue with a dual function, being activated during cold exposure and also during overfeeding [[14] and references therein]. Given the importance of D2 in normal BAT recruitment, it is surprising that D2KO mice do not have a phenotype reflecting a significant disruption of energy balance. Both increased and decreased weight gain have been associated with models of BAT dysfunction. For example, mice with a targeted ablation of BAT due to a tissue-specific transgenic expression of diphtheria toxin from the uncoupling protein 1 (UCP1) gene promoter are obese, although it is possible that the obesity of this mouse model is caused by hyperphagia rather than reduced thermogenesis [15]. Additional mouse models with impaired BAT function such as mice with a deletion of all 3 β-adrenergic receptors, and knock in of a dominant negative mutation in TRα (P398H), have increased weight gain even on a chow diet [16], [17]. On the other hand, other models of BAT dysfunction have a paradoxical resistance to obesity such as mice with targeted deletion of the UCP-1 gene, or deletion of all isoforms of TRα (*Thra-0/0)*, or a knock in of a different dominant negative mutation in TRα (R384C) [18]–[20]. In these models it is thought that BAT impairment leads to alternative, more energetically costly forms of facultative thermogenesis, resulting in a resistance to obesity. In support of this, the reduced sensitivity to obesity in these mouse models is attenuated as ambient temperature is increased, and disappears at thermoneutrality where there is no need to defend core temperature [18]–[20].

In this work we report studies where the weight gain of D2KO mice on a high fat diet (HFD) was compared to congenic wild type controls, and we find that there is a modest, but significant, increase in weight gain in D2KO mice. In addition, our data indicates that D2KO mice are insulin resistant on a chow diet, even without increased weight gain. We conclude that the loss of the *Dio2* gene results in significant metabolic consequences.

## Methods

### Animal Treatment Protocols

Animals were maintained and experiments were performed according to protocols approved by the Animal Care and Use Committee of Harvard Medical School and Baystate Medical Center. D2KO mice were generated as previously described [4] in collaboration with Drs. Donald St. Germain and Valerie Galton. The D2KO mice used in this study were backcrossed for 11-generations with C57BL/6 mice from Jackson Labs (Bar Harbor, ME) and can be considered congenic with C57BL/6 mice except for the targeted disruption of the *Dio2* gene [21]. Control animals were also derived from C57BL/6 mice from Jackson Labs, and were bred, born, and raised in our animal facility in parallel with D2KO mice. For energy expenditure experiments control mice were purchased from Jackson Labs, and were maintained in parallel in the same animal facility 2 weeks before use. Under these conditions D2KO showed a similar increase in weight gain when on a HFD (data not shown). Mice were maintained for 6 weeks on either a control diet of standard chow (# 7001) or 21% milk fat diet (48% of calories derived from fat) (# 95121) from Harlan Teklad as described [22]. Mice were 9-weeks old at the start of the diet, with average starting weights being 22.8±0.3 g and 22.5±0.3 g for wild type and D2KO mice on a HFD, while starting weights were 21.8±0.6 g and 22.8±0.3 g for wild type and D2KO mice on a chow diet.

### Glucose and Insulin Tolerance Testing

Glucose and insulin tolerance testing were performed as described previously [23]–[25]. Briefly, for glucose tolerance testing mice were fasted 14 h, and injected intra-peritoneally with 2 g/kg D-glucose. 0 to 120 minutes after injection, glucose levels were measured using a glucometer (One Touch Ultra, Lifescan) in blood collected from the tail vein. Blood was also collected at 0, 30 and 120 minutes from the same mice for measurement of insulin levels. For insulin tolerance testing, mice were fasted 14 h, then injected with intra-peritoneally with 0.5 mU/g body weight. Blood glucose levels were then measured from 0 to 120 minutes after injection, as in glucose tolerance testing. The HOMA-IR index was calculated as described [26].

### Deiodinase Activity

D2 assays were performed as described [27], [28]. Muscle D2 was measured by assaying 75–100 µg of microsomal protein, while BAT D2 activity was measured by assaying 50 µg of homogenate for 3 hours at either 1 nM (specific) or 100 nM T4 (non-specific background).

### qRT-PCR

Quantitative Real-Time PCR (qRT PCR) was performed as described previously [29] with the following modifications: SuperScript VILO (Invitrogen, Carlsbad CA) was used for cDNA synthesis following the manufacturer's instructions. Sequences of primers used are listed in Table S1. All samples were normalized for the amount input of mRNA using Cyclophilin A expression, a commonly used housekeeper that we have found to be independent of thyroid status [28].

### Serum Hormone/Chemistry Measurements

Levels of total serum T3 and T4 were measured as described previously [29]. Serum triglyceride and glycerol levels were determined using a serum triglyceride measurement kit from Sigma, while β-hydroxybutyrate and free fatty acid levels were measured using colorimetric assays at the Joslin Diabetes Center Specialized Assay Core. Serum insulin levels were measured using an Insulin EIA kit (ALPCO).

### Liver Triglyceride Content

Liver triglycerides were extracted by incubating 100 mg of minced liver with 15 ml of 1∶2 methanol: chloroform and 3 ml of 0.05% H_2_SO_4_ overnight at room temperature. 1 ml of the lower phase was transferred to a new tube and 0.5 ml chloroform +1% triton X-100 added, and samples were dried using nitrogen. Samples were then resuspended in 1–3 ml water as needed and triglycerides were measured using a triglyceride quantification kit (Abcam, Cambridge MA) following the manufacturer's instructions for fluorometric assay.

### Liver Glycogen Content

Glycogen content was determined *via* acid hydrolysis followed by measurement of glucose [30], [31]. In brief, ∼10 mg of liver was homogenized in 0.5 ml 2 N HCl, boiled for 2 hours, and neutralized with an equal volume of 2 N NaOH. Samples for background glucose determination were homogenized in 0.5 ml 0.03 N HCL, boiled for 5 minutes, and neutralized by an equal volume of 0.03 N NaOH. Glucose content was measured using glucose (hexokinase) assay reagent from Sigma (St. Louis, MO).

### Measurement of Body Composition by MicroCT

Body Composition was measured at the Longwood Small Animal Imaging Facility of Beth Israel Deaconess Medical Center. Mice were anesthetized with 2% isoflurane/balance O_2_. Imaging was performed using the CT component of a NanoSPECT/CT (Bioscan, Washington, DC) scanner equipped with an 8 W X-ray source running at 45 kVp (177 mA) and a 48 mm pitch CMOS-CCD X-ray detector. Continuous helical micro-CT scanning was employed with the following parameters: 1 s exposure, 240 angles, 1.3 magnification, 37 mm pitch (1 field-of-view), and a 512×256 pixel frame size (192 mm pixels). Images were reconstructed as 170×170 pixel transverse matrices with varying axial length and slice thickness of 0.1 mm (isotropic voxel size 0.1 mm) using filtered-back projection (Shepp-Logan filtering). Quantitation of body fat was performed using InVivoScope software (Bioscan, Inc.)

### Measurement of V0_2_ and RER

Energy expenditure was measured as described previously by indirect calorimetry using an open-circuit system (Qubits System, Kingston, Ontario Canada) [19]. Measurements were done over a 24-hour period, and begun at mid-afternoon after mice had several hours to adjust to the chambers. Mice had *ad libitum* access to food and water during the course of experiments, and there was no significant change in the weight of mice under these conditions.

### Statistical Analysis

Prism 4.0 software (GraphPad Software, San Diego CA) was used for statistical analysis. When only two groups were analyzed, statistical significance was determined using an unpaired Student's t-test. Two-way ANOVA was used to compare the effects of different diets (chow and HFD) on two genotypes (WT and D2KO), and when significant differences were observed individual means of columns were compared by unpaired Student's t-test as indicated. Repeated measure based parameters (such as weight gain over time or GTT) were analyzed using two-way ANOVA for repeated measures followed by Bonferroni correction. Statistical details (p-value, F, and degree of freedom (Df)) are provided in the figure legends along with the results of the two-way ANOVA testing. p<0.05 was considered statistically significant.

## Results

### D2KO mice gain more weight on a high fat diet

**Figure 1:**
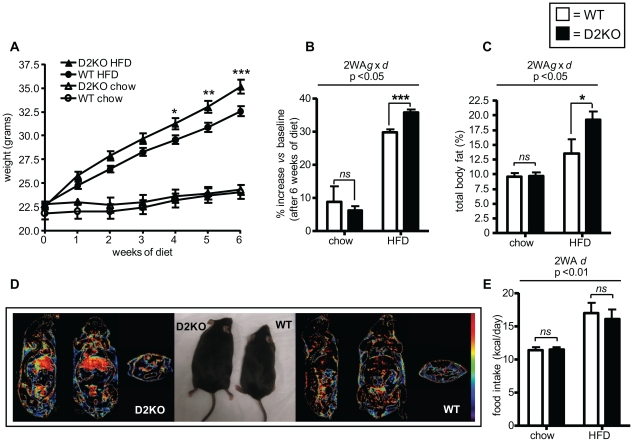
Male D2KO mice weight gain and body fat on a HFD. (A) Body weight of wild type and D2KO mice on chow or a HFD for 6-weeks, n = 22–24 mice/genotype for HFD group, n = 5 mice/genotype for chow group. A significant interaction between genotype and diet were determined by two-way ANOVA for repeated measures (p<0.001; F = 33.55; Df = 312). After Bonferroni correction, there was a significant difference in weight gain of D2KO vs. WT after 4, 5 and 6 weeks of diet (p<0.05, 0.01 and 0.001). (B) Weight gain expressed as % of initial weight after 6 weeks on either chow or a HFD of mice shown in (A). Two-way ANOVA indicated a significant interaction between genotype and diet (2WA g×d) (p<0.05; F = 7.11; Df = 52). (C) % body fat as determined by microCT, n = 4–5 mice/group. Two-way ANOVA indicated a significant interaction between genotype and diet (p<0.05; F = 4.87; Df = 15). (D) Density of voxels falling in the Hounsfield Unit range defined for adipose tissue of a representative wild type and D2KO mouse. Red is more dense while purple is less in the colorbar range. (E) Average food intake of wild type and D2KO mice on either a chow or HFD was monitored for 7 days at 3 weeks of diet and is shown expressed as kcal/day/mouse. Two-way ANOVA indicated that diet significantly effected the overall caloric consumption independent of genotype (2WA d) (p<0.01; F = 10.25; Df = 16). When a significant interaction between genotype and diet was found individual means were compared within groups by unpaired Student's t-test. Data presented are the mean ± SEM; * = p<0.05, ** = p<0.01, *** = p<0.001, ns = not significant.

We first compared weight gain of D2KO mice backcrossed 11-generations in a C57BL/6 background with control C57BL/6 mice on a chow and high fat diet containing 21% milk fat (HFD) for 6-weeks (Fig. 1A). While male wild type and D2KO mice both gained similar amounts of weight on a chow diet, notably, D2KO mice gained 5.6% more weight than control animals (p<0.001) (Fig. 1A,B). Analysis of the percentage of body fat using micro CT confirmed no difference between genotypes on chow; however, D2KO mice had 28% more body fat than wild type animals when on a HFD (p<0.05) (Fig. 1C,D). Despite their increased weight gain, D2KO mice did not eat more than control animals, with WT and D2KO mice having the same caloric intake on either diet (Fig. 1E). Similar results were found when female mice were studied with D2KO female mice gaining more weight on HFD than WT mice and having with 43% more body fat after 6 weeks of HFD (p<0.001) (Fig. S1).

**Figure 2:**
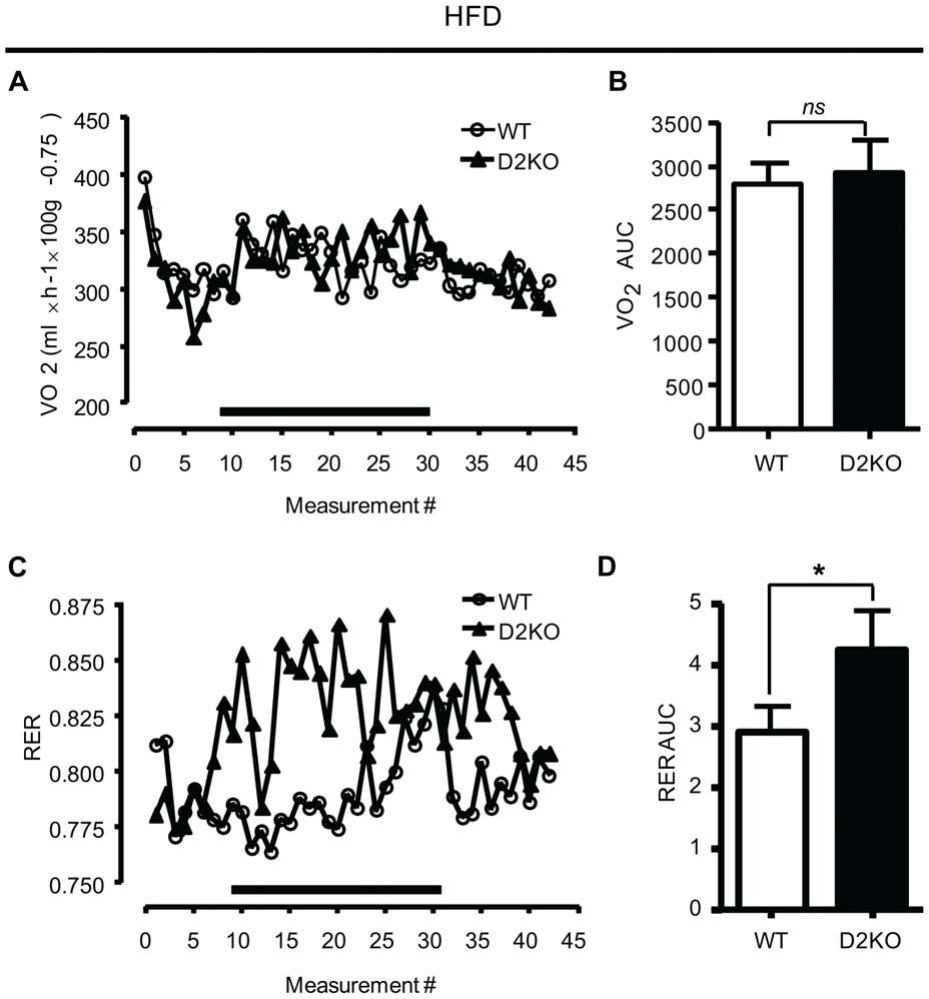
VO_2_ consumption and RER of wild type and D2KO mice on a HFD. (A) 24-hour VO_2_ profile of wild type and D2KO mice on a HFD. Each point represents the mean of 5 mice measured every 35 minutes. The black bar below the X-axis represents the dark period during which mice would be feeding. (B) Histograms representative of the areas under the curve of (A). (C) 24-hour RER of wild type and D2KO mice on a HFD. Each point represents the mean of 5 mice measured every 35 minutes. (D) Histograms representative of the areas under the curve of (C) Data presented are the mean ± SEM; * = p<0.05, ns = not significant.

On a chow diet no difference in oxygen consumption (VO_2_) or respiratory exchange ratio (RER) was observed between genotypes (data not shown). On a HFD, VO_2_ was not different between genotypes, but RER was greater in the D2KO genotype (p<0.05), indicating that the relative contribution of carbohydrate to oxidative metabolism is increased (Fig. 2 A, B, C, D). Notably, during darkness (indicated by the bar), when rodents eat, the difference between the average RER is even more marked, being 0.82±0.003 versus 0.80±0.004 (p<0.001). Applying the Lusk equation [32], this means that D2KO were burning 39% of carbohydrate and 61% of fat, whereas the WT burned 31% carbohydrate and 69% fat (p<0.001) during this period, despite both being on the same diet.

### D2KO mice are insulin resistant even on a chow diet

**Figure 3 pone-0020832-g003:**
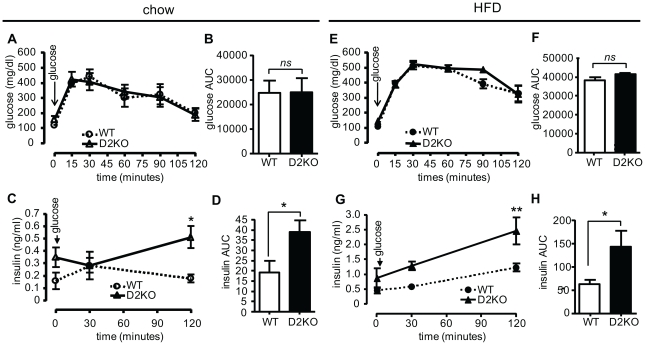
Glucose tolerance testing and corresponding insulin levels of wild type and D2KO mice on a chow and HFD. (A) Wild type and D2KO mice maintained on a chow diet were fasted for 14 h, and then injected IP with 2 g/kg D-glucose at time 0 indicated by the arrow. Blood glucose levels were measured at the indicated time, and are not significantly different by two-way ANOVA for repeated measures. (B) Histograms representative of the areas under the curve of (A). (C) Corresponding serum insulin levels of mice in (A). A significant difference was found by two-way ANOVA for repeated measures after Bonferroni correction at 120′ (p<0.05). D) Histograms representative of the areas under the curve of (C). (E) Same as (A) except mice were maintained for 6-weeks on a HFD prior to testing, values are not significantly different by two-way ANOVA for repeated measures. (F) Histograms representative of the area under the curve of (E). (G) Corresponding serum insulin levels of mice in (E). A significant interaction was found by two-way ANOVA for repeated measures (p<0.05; F = 4.26, Df = 16). A significant difference was found after Bonferroni correction at 120′ (p<0.01). (H) Histograms representative of the area under the curve of (G). N = 5–6 mice/group, male mice were 15 weeks old time of at testing. Data shown are the mean ± SEM with * = p<0.05, ** = p<0.01, ns = not significant.

Since increased body fat is associated with insulin resistance, we assessed if the greater weight gain of D2KO mice would lead to differences in glucose handling and insulin responsiveness. Fasting blood glucose levels were significantly higher in D2KO mice on chow (p<0.01), while corresponding insulin levels showed a tendency to be higher (Table 1). Accordingly, the HOMA index was also higher in D2KO mice (p<0.05). When glucose tolerance testing was performed on male chow-fed D2KO and control mice glucose clearance was not different between genotypes, however insulin levels in the D2KO group were 2.5-times that of wild type animals 120 minutes after glucose injection (p<0.05) (Fig. 3A,B,C,D). When the HFD groups were studied, D2KO mice had significantly higher fasting glucose and insulin levels (p<0.01 and p<0.05) with an increase in their HOMA index (p<0.01) (Table 2). With GTT, overall the glucose clearance between D2KO and wild type animals appeared similar (Fig. 3E,F). Notably, after glucose injection the increase in insulin levels in D2KO mice was much greater, being double that of controls at 120 minutes (p<0.01) (Fig. 3G,H). Results of glucose tolerance testing in HFD fed female D2KO mice were similar to those of males, with no difference in glucose levels over a time course of 120 minutes (Fig. S2A,B) while insulin levels were significantly higher at both 30 and 120 minutes (Fig. S2C,D).

**Table 1:** Fasting glucose and insulin levels of WT and D2KO mice on chow diet.

	WT	D2KO	
*n*	10	10	P value
fasting glucose (mg/dL)	82.1±2.2	95.6±3.5	<0.01
fasting insulin (ng/mL)	0.35±0.04	0.48±0.06	ns
HOMA index	1.75±0.21	2.85±0.38	<0.05

Mean ± SEM is shown. Male WT and D2KO mice were 9 week old at time of testing. HOMA index was calculated as fasting glucose (mg/dl) x fasting insulin(µU/mL)/405 as in [26].

**Table 2:** Fasting glucose and insulin levels of WT and D2KO mice on high fat diet (HFD).

	WT	D2KO	
*n*	16	16	P value
fasting glucose (mg/dL)	102.0±6.0	130.8±7.6	<0.01
fasting insulin (ng/mL)	0.28±0.05	0.53±0.1	<0.05
HOMA index	1.86±0.33	4.10±0.72	<0.01

Mean ± SEM is shown. Male WT and D2KO mice were 15–16 weeks old and on HFD for 6 weeks, at the time of testing. HOMA index was calculated as fasting glucose (mg/dl) x fasting insulin(µU/mL)/405 as in [26].

**Figure 4:**
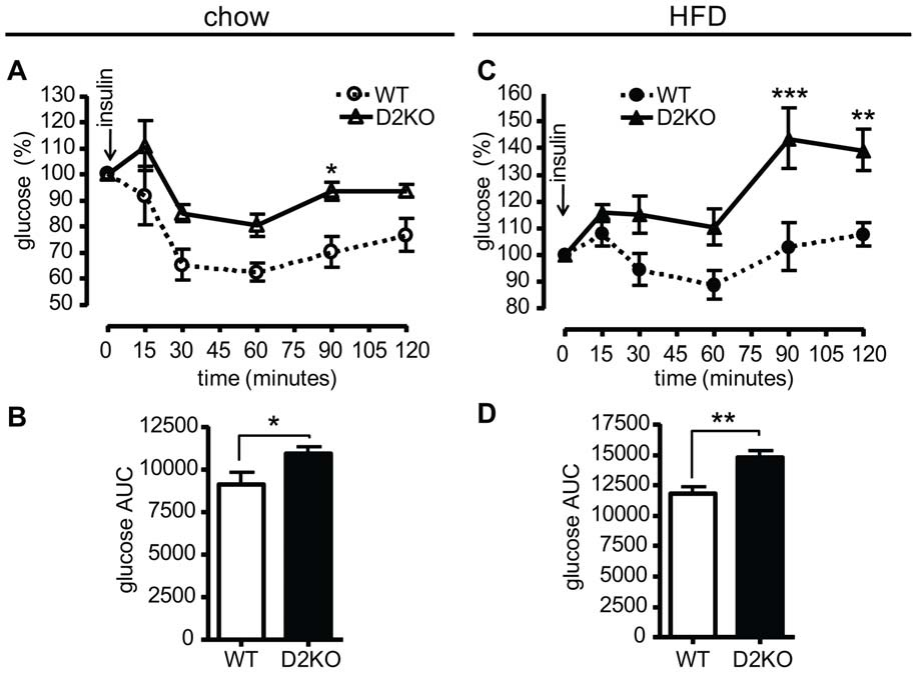
Insulin tolerance testing of wild type and D2KO mice on chow and HFD. (A) wild type and D2KO mice maintained on a chow diet were fasted for 14 h, and then injected IP with 0.5 mU/g body weight insulin at time 0 indicated by the arrow. A significant difference was found by two-way ANOVA for repeated measures after Bonferroni correction at 90′ (p<0.05). (B) Histograms representative of the area under the curve of (A). (C) Same as (A) except mice were maintained for 6-weeks on a HFD prior to testing. Two-way ANOVA for repeated measures showed a significant interaction (p<0.05; F = 3.09; Df = 55). Significant differences were found after Bonferroni correction at 90′ and 120′ (p<0.001 and p<0.01) (D) Histograms representative of the area under the curve of (C). N = 5–7 mice/group, male mice were 15 weeks old time of at testing, data shown are the mean ± SEM with * = p<0.05, ** = p<0.01, *** = p<0.001.

The above results indicate that D2KO mice require greater amounts of insulin to normalize their serum glucose levels suggestive of insulin resistance, and thus we performed insulin tolerance testing to confirm this. Glucose levels were significantly higher in D2KO mice on a chow diet at 90 minutes after a single i.p. injection of insulin (p<0.05), with the integrated area under the curve also being significantly greater in D2KO mice (p<0.05) (Fig. 4A,B). D2KO mice on a HFD have significantly higher blood glucose levels after insulin injection with values being significantly increased at 90 and 120 minutes (p <0.001 and 0.01), with the integrated area under the curve also indicating that D2KO mice have a decreased response to insulin (p<0.01) (Fig. 4C,D). Furthermore, D2KO mice on a HFD showed a paradoxical rise in blood glucose levels that was particularly evident at late times after insulin injection. Taken together these results indicate that the D2KO mice have abnormal glycemic control, and peripheral resistance to the action of insulin. Remarkably, these defects are apparent when mice are on a chow diet, even before increased weight gain.

### Assessment of serum and liver biochemistry

**Figure 5:**
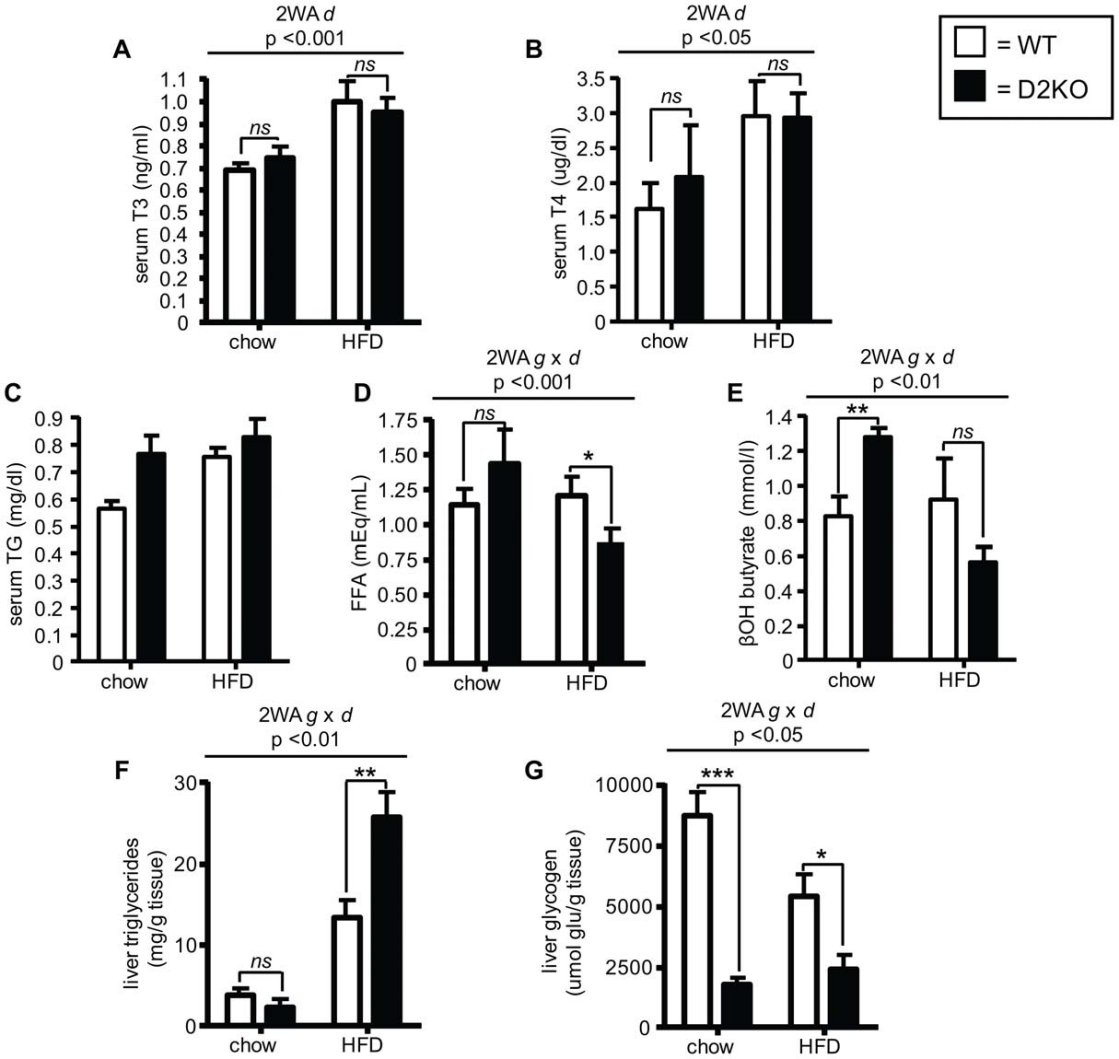
Serum and liver biochemistry of wild type and D2KO mice on chow and HFD. Levels of T3 (A) T4 (B), (C) triglycerides, (D) free fatty acids, or (E) β-hydroxybutyrate in serum are shown. Hepatic levels of (F) triglycerides or (G) glycogen are also indicated. Data are analyzed by two-way ANOVA. There was a significant effect of diet (2WA d) on T3 (p<0.001; F = 16.45; Df = 19) and T4 (p<0.05; F = 4.73; Df = 20) independent of genotype. Two-way ANOVA indicated a significant interaction between diet and genotype (2WA g×d) for serum FFA (p<0.001; F = 4.91; Df = 20); β-hydroxybutyrate (p<0.01; F = 8.55; Df = 19), hepatic triglycerides (p<0.05; F = 6.31; Df = 19) and hepatic glycogen (p<0.05; F = 5.37; Df = 20). When a significant interaction between genotype and diet was found individual means were compared within groups by unpaired Student's t-test and showed in the figure. Data shown are the mean ± SEM of 4–5 mice/group on chow diet or 7–10 mice/group on HFD with * = p<0.05, ** = p<0.01, *** = p<0.001, ns = not significant.

To define the metabolic consequences of HFD feeding in D2KO mice, we first determined levels of relevant serum markers. As shown previously, serum T3 values were not different between wild type and D2KO animals, while T4 had a tendency to be increased in D2KO mice on a standard chow diet [4], [27] (Fig 5A,B). Diet had a positive effect on both serum T3 and T4 independent of genotype, with both being increased after 6 weeks of HFD to similar extents in both wild type and D2KO mice (p<0.001 and p<0.05 by two-way ANOVA) (Fig 5A,B). Triglyceride levels were not significantly different between genotypes and did not change with diet, although they had a tendency to be elevated in D2KO mice on a chow diet (Fig. 5C). FFA levels were lower in D2KO mice on a HFD when compared to the control animals (p<0.05) (Fig. 5D), while β-OH-butyrate was increased in D2KO mice on chow when compared to controls (p<0.01) (Fig. 5E).

In the liver, triglyceride content was increased by 57% in D2KO mice on a HFD (p<0.01) (Fig. 5F). Strikingly, glycogen content was significantly decreased by greater than 60–80% in D2KO mice on both chow and a HFD (p<0.001 and p<0.05) (Fig. 5G). Overall, the above results are consistent with a preferential use of carbohydrates/impairment of fatty acid utilization in D2KO mice on a HFD.

### Gene expression patterns in liver, muscle, and brown and white adipose tissue of wild type and D2KO mice on chow and a HFD

**Figure 6:**
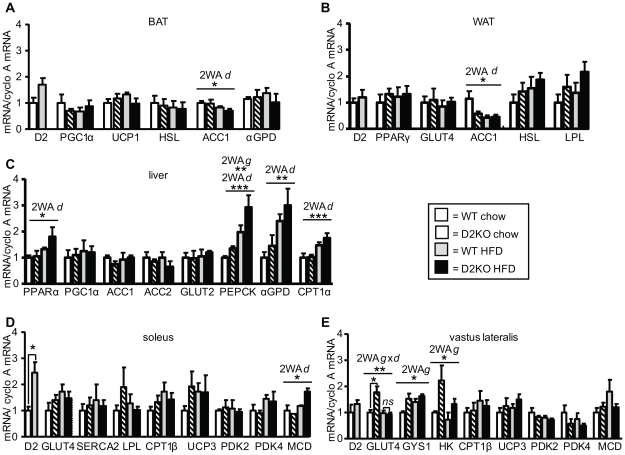
Levels of gene expression in wild type and D2KO mice on chow and HFD. mRNA levels of the indicated genes were measured using qRT PCR, and then corrected by Cyclophillin A expression as a house-keeping gene. Data is normalized to expression of wild type mice on a chow diet, and expression levels in (A) BAT, (B) WAT, (C) liver, (D) soleus, and (E) vastus lateralis are shown. Significant effects were determined by two-way ANOVA. A significant interaction between genotype and diet (2WA g×d) was found in vastus lateralis for GLUT4 (p<0.01; F = 8.79; Df = 16). A significant effect of diet (2WA d) independent of genotype was found in BAT for ACC1 In BAT (p<0.05; F = 6.02; Df = 16), in WAT for ACC1 (p<0.05; F = 5.41; Df = 16), in liver for PPARα(p<0.05; F = 6.0; Df = 16), PEPCK (p<0.001; F = 30.19; Df = 16), αGPD (p<0.01; F = 12.21; Df = 16), CPT1α (p<0.001; F = 21.87; Df = 16), and in soleus for MCD (p<0.01; F = 6.95; Df = 8). A significant effect of genotype (2WA g) independent of diet was found in liver for PEPCK (p<0.01; F = 8.91; Df = 16) and in vastus lateralis for GYS1 (p<0.05; F = 8.02; Df = 16) and HK (p<0.01; F = 5.9; Df = 16). When a significant interaction between genotype and diet was found individual means were compared within groups by unpaired Student's t-test. N = 5 mice/group except for soleus were n = 3–4 with each sample being solei pooled from 3 mice. Data shown are the mean ± SEM; * = p<0.05, ** = p<0.01, *** = p<0.001, ns =  not significant.

Expression levels of genes relevant to glucose and fatty acid metabolism were measured in brown and white adipose tissue, liver, and muscle of wild type and D2KO mice on both diets (Fig 6A–F). While numerous genes changed in both genotypes in response to HFD feeding, no obvious differences were observed that could explain the increased weight gain of D2KO mice on a HFD. In BAT no significant changes in mRNA levels were found, beside the reduction of acetyl CoA carboxylase 1 (ACC1) expression when mice were on a HFD independent of genotype (p<0.5 by two-way ANOVA) (Fig. 6A). However, in wild type mice, D2 gene expression showed a tendency to be increased by HFD feeding in this tissue (Fig. 6A), correlating with a 2.9-fold increase in D2 activity (p<0.05) (data not shown). In white adipose tissue, ACC1 was also significantly decreased by HFD feeding independent of genotype (p<0.5 by two-way ANOVA) (Fig. 6B).

In liver, peroxisome proliferator activated receptor α (PPARα), carnitine palmitoyltransferase 1 α (CPT1α), and α-gylcerolphosphate dehydrogenase (αGPD) were all increased when mice were on a HFD, independent of the genotype (p<0.05, p<0.01, and p<0.001 by two-way ANOVA) (Fig. 6C). Levels of PEPCK, a marker of gluconeogenesis, increased when mice were fed a HFD (p<0.001 by two-way ANOVA), and further were increased to a greater extent in D2KO mice (p<0.01 by two-way ANOVA), consistent with the increased serum glucose levels found in D2KO mice (Fig. 6C, Table 1, Table 2). Previously we have found low D2 mRNA expression and activity in liver of mice and rats that can be modulated by diet, however no D2 mRNA or activity were detectable in these experiments [22], [33].

In soleus there were no striking changes in gene expression between either genotype on chow or HFD, although the expression of malonyl CoA decarboxylase (MCD) was significantly increased by HFD feeding for both genotypes (Fig. 6D) (p<0.05 by two-way ANOVA). D2 mRNA was increased 2.5-fold by a HFD in the soleus of wild type mice (p<0.05) (Fig. 6D), although D2 activity remained unchanged (data not shown). In vastus lateralis muscle genotype significantly effected the expression of glycogen synthetase 1 (GSY1) and hexokinase (HK), with levels of these genes being greater in D2KO mice independent of diet (Fig. 6E) (p<0.05 by two-way ANOVA). Glucose transporter 4 (GLUT4) was also increased in chow-fed D2KO mice (p<0.05), while this difference was no longer apparent when D2KO mice were on a HFD.

## Discussion

In these studies we report that D2KO mice gained 5.6% more weight than wild type mice when challenged for 6-weeks with a HFD (Fig. 1A,B). Although this represents a modest percentage increase, body fat of D2KO mice was increased by 28% when compared to wild type mice (Fig. 1C,D). Notably, previous studies have shown that even a small amount of weight loss and decrease in adiposity can significantly decrease the risk of developing diabetes and cardiovascular disease [34]–[37].

Surprisingly, the increased weight gain of D2KO mice is not the result of increased food intake, or decreased energy expenditure since VO_2_ consumption was the same between both genotypes when mice had *ad libitum* access to food. Interestingly, in a subsequent set of experiments, during fasting D2KO showed a significant reduction in VO_2_ consumption as compared to wild type controls, suggesting that D2KO mice do have reduced energy expenditure under some conditions [38]. RER measurement indicates that D2KO mice burn ∼8% more carbohydrate, and correspondingly less fat, despite their apparent insulin resistance (Fig. 2–4, Table 1, Table 2). Additionally, serum levels of free fatty acids and β-hydroxybutyrate were lower in D2KO mice on a HFD, indicating that fatty acid mobilization/oxidation might be impaired. Consistent with these findings, hepatic triglycerides were increased in D2KO mice fed a HFD, while glycogen levels are lower despite increased PECK suggestive of increased gluconeogenesis (Fig. 5D–G, Fig. 6C). Taken together, our results suggest that on a HFD D2KO mice may have an increased reliance on carbohydrate as a fuel source with a tendency to accumulate the fat that is not utilized, and that this in turn could be one of the factors contributing to the increased weight gain of D2KO mice on a HFD. Pyruvate kinase dehydrogenases (PDKs) are key regulators of glucose oxidation *via* phosphorylation of the pyruvate dehydrogenase complex, and mice with a targeted deletion of PDK 4 exhibit a preferential usage of glucose in muscle [39], [40]. Both PDK 2 and 4 are T3-responsive genes, and expression of some, but not all, T3-responsive genes has been found to be impaired in D2KO mice [8], [13], [41]–[43]. However, PDK2 and 4 are mRNA levels are unchanged in D2KO mice, and the molecular basis of preferential carbohydrate usage in D2KO mice remains to be defined (Fig. 6D,E).

D2 activity has been described in BAT, white adipose tissue, muscle, liver and brain [22], [33], [44]–[47]. Due to the global loss of D2 in our model, the absence of D2 in any of these tissues could contribute to the increased weight gain of the D2KO mice. However, D2 activity was unmeasurable in liver and white adipose tissue of our wild type mice in these experiments, thus any effects observed in these tissues are most likely indirect.

The hypothalamus is a D2 containing tissue that plays a key role in energy homeostasis (reviewed in [2], [48]). Hypothalamic D2 has been shown to be important in UCP-2 mediated re-feeding behavior, and D2KO mice consume less food immediately after fasting [49]. However our results indicate that under conditions of *ad libitum* feeding alterations in food intake did not contribute to the increased weight gain found in D2KO mice (Fig. 1 E). AMPK in the hypothalamus is also T3-regulated, with central administration of T3 in rats decreasing AMPK activity [50]. This in turn leads to increased sympathetic nervous system activity and BAT stimulation, resulting in an up-regulation of thermogenic markers and weight loss. With this in mind, a loss of D2 might result in impaired local generation of T3 from T4 in the ventromedial nucleus of the hypothalamus in D2KO mice, resulting in decreased tissue-T3 content. This could lead to increased AMPK activity and decreased sympathetic nervous system activity, resulting in impaired activation of brown adipose tissue (BAT) favoring increased weight gain. Furthermore, decreased sympathetic stimulation of the liver might also lead to increased hepatic triglyceride accumulation. However, in this regard, D2KO mice have increased norepinephrine turnover in BAT, indicating increased sympathetic activity, suggesting that the mechanism detailed above is not the explanation for their increased weight gain [13], [51]. Additionally, while the increased hepatic triglyceride levels found in D2KO mice could be a result of decreased sympathetic stimulation of the liver, on the other hand, their increased levels of PEPCK, suggesting increased gluconeogenesis, and decreased glycogen content, are consistent with increased sympathetic stimulation (Fig. 5 F,G, Fig. 6C) [52]–[54]. Thus, there are likely to be multiple factors contributing to the hepatic phenotype of the D2KO mice.

D2 activity is increased by 2.9-fold in the BAT of wild type mice with HFD feeding, consistent with the increased activation of this tissue found during diet induced thermogenesis. Given the key role BAT plays in energy expenditure, it seems likely that BAT dysfunction could be contributing to the increased weight gain found in D2KO. Indeed, many mouse models show an inverse relationship between D2 in BAT and adiposity [18], [19], [55]. Further, an increase in D2 in BAT (and potentially other tissues) is necessary for bile acid supplementation to increase energy expenditure in mice fed a HFD, thus conferring resistance to diet induced obesity to these animals [56]. However, it should be noted, that whatever the mechanism behind the increased weight gain of D2KO mice, it is not simply the result of impaired BAT function leading to decreased oxygen consumption (Fig. 2). In this regard, increased D2 expression in liver, muscle, and WAT has also been described in other models of resistance to weight gain, although the significance of this has yet to be fully understood [18], [19], [22], [57]. While our data suggests that a loss of D2 in liver and WAT may not be relevant in our model, a loss of D2 in skeletal muscle could result in significant consequences, given the major role played by this tissue in glucose disposal and fuel consumption [58].

In spite of their preference for carbohydrate usage, D2KO mice have increased resistance to insulin when on a HFD as denoted by greater insulin levels during GGT, a decreased glucose uptake during ITT, and an increased HOMA index, as might be expected due to their increased adiposity (Fig. 3, 4, Table 2). However, it is remarkable that even at 9-weeks of age when on a chow diet, D2KO mice have higher fasting glucose levels and an increased HOMA index (Table 1), and also have greater insulin levels during GGT and a decreased glucose uptake during ITT (Fig. 3, 4), all consistent with insulin resistance of D2KO even prior to increased weight gain. This phenotype is in agreement with other work showing that primary cultures of both brown adipocytes and myocytes lacking D2 have reduced levels of Akt phosphorylation after insulin stimulation [43], [59]. Additionally, increased triglyceride accumulation in liver, as found in the D2KO mice, has been correlated with insulin resistance (Fig. 5F)[60]. Gene expression profiles further indicate that D2KO mice have higher levels of PEPCK in liver, and this difference is magnified with high fat diet feeding, suggesting that increased gluconeogenesis could play a role in the increased fasting glucose levels found in the D2KO mice (Fig. 6C). With this in mind, we cannot rule out that the higher insulin levels found in D2KO mice may in part be due to compensation for increased gluconeogenesis. Nonetheless, D2KO mice exhibit alterations in glucose homeostasis that are consistent with a pre-diabetic state [61].

In humans, D2 has been linked to insulin resistance, with a polymorphism in the human *Dio2* coding region (Thr92Ala) found at a frequency of 0.32 in the general population, and 0.75 in Pima Indians, being associated with insulin resistance in some studies [62]–[66]. In line with this, hypothyroidism has also been associated with insulin resistance that can be improved with thyroid hormone treatment [67]. Our work indicating that D2KO mice are insulin resistant even before increased weight gain would further support a role for D2 in the modulation of insulin sensitivity.

While this paper was under review, other studies were published by Castillo et al. detailing the effects of HFD feeding on D2KO mice [51]. In contrast to our results, this work did not find increased weight gain in D2KO mice fed a HFD at room temperature, while D2KO weight gain with HFD feeding was increased when mice were maintained at thermoneutrality to minimize the effects of increased sympathetic tone. We speculate that the closer match between the starting weights of our mice (22.8 g wild type and 22.5 g D2KO versus ∼26 g wild type and ∼21 g D2KO) and a greater number of animals in our study (22–24/genotype versus 4–5/genotype) may have allowed us to uncover what otherwise might have been a subtle phenotype. Castillo et al. also report that chow-fed D2KO mice have increased glucose clearance during GTT that is lost with HFD feeding, with no data on the corresponding insulin levels in these mice [51]. However, our results are more consistent with other work from the same group that found that no difference in glucose clearance during GTT between chow-fed wild type and D2KO mice [68]. Lastly, both our groups find no change in VO_2_ in HFD fed D2KO mice at room temperature, and an elevation of hepatic triglyceride content [51].

In summary, our results indicate that a loss of the *Dio2* gene in mice results in greater weight gain, hepatic steatosis on a HFD, and insulin resistance even before D2KO mice have increased adipose tissue. Further, D2 may play an important role in regulating intermediary metabolism and fuel partition. Taken together as a whole, our data suggests that D2 could be an important target in terms of modulation of adiposity, and in the regulation of insulin action.

## Supporting Information

Figure S1 **Female D2KO mice weight gain and body fat on a HFD.** (A) Body weight of wild type and D2KO mice on chow or a HFD for 6-weeks. n = 4–5 mice/group. A significant interaction between genotype and diet was determined by two-way ANOVA for repeated measures (p<0.001; F = 21.02; Df = 96). After Bonferroni correction, there was a significant difference in weight gain of D2KO vs. WT starting at week 2 (B) Weight gain expressed at % of initial weight after 6 weeks on either chow or a HFD of mice shown in (A), two-way ANOVA indicated a significant interaction between genotype and diet (2WA g×d) (p<0.05; F = 7.52; Df = 16). (C) Weight of dissected perigonadal, mesenteric, perirenal, subcutaneous and brown adipose tissue fat depots divided by total body weight was used to calculate adiposity index as in [18], two-way ANOVA indicated a significant interaction between genotype and diet (2WA g×d) (p<0.0001; F = 37.52; Df = 16). (D) Average food intake of wild type and D2KO mice on either a chow or HFD was monitored for 7 days at 3 weeks of diet and is shown expressed as kcal/day/mouse. Two-way ANOVA indicated diet significantly effected the overall caloric consumption independent of genotype (2WA d) (p<0.001; F = 15.41; Df = 16). Data shown are the mean ± SEM, * = p<0.05, ** = p<0.01, *** = p<0.001, ns = not significant.
(TIF)

Figure S2 **Glucose tolerance testing and corresponding insulin levels of female wild type and D2KO mice on a HFD.** Female wild type and D2KO mice were maintained for 6-weeks on a HFD prior to testing. (A) Results of glucose tolerance testing performed as in Fig. 3A are shown. Blood glucose levels were measured at the indicated time, and are not significantly different by two-way ANOVA for repeated measures. (B) Histograms representative of the area under the curve of (A). (C) Corresponding serum insulin levels of mice in (A) Two-way ANOVA for repeated measures showed a significant effect (p value<0.01; F = 8.15, Df = 16). After Bonferroni correction significant differences were found at 90′ and 120′ (p<0.05 and p<0.001) (D) Histograms representative of the area under the curve of (C). N = 4–5 mice/group, female mice were 15 weeks old time of at testing. Data shown are the mean ± SEM with * = p<0.05, ** = p<0.01; ns = not significant.
(TIF)

Table S1 (DOC)
