# Supplementary figures and images for: Mice with a Targeted Deletion of the Type 2 Deiodinase Are Insulin Resistant and Susceptible to Diet Induced Obesity
Source: PLoS One. 2011 Jun 16;6(6):e20832. doi: 10.1371/journal.pone.0020832 (PMC3116839; doi:10.1371/journal.pone.0020832)

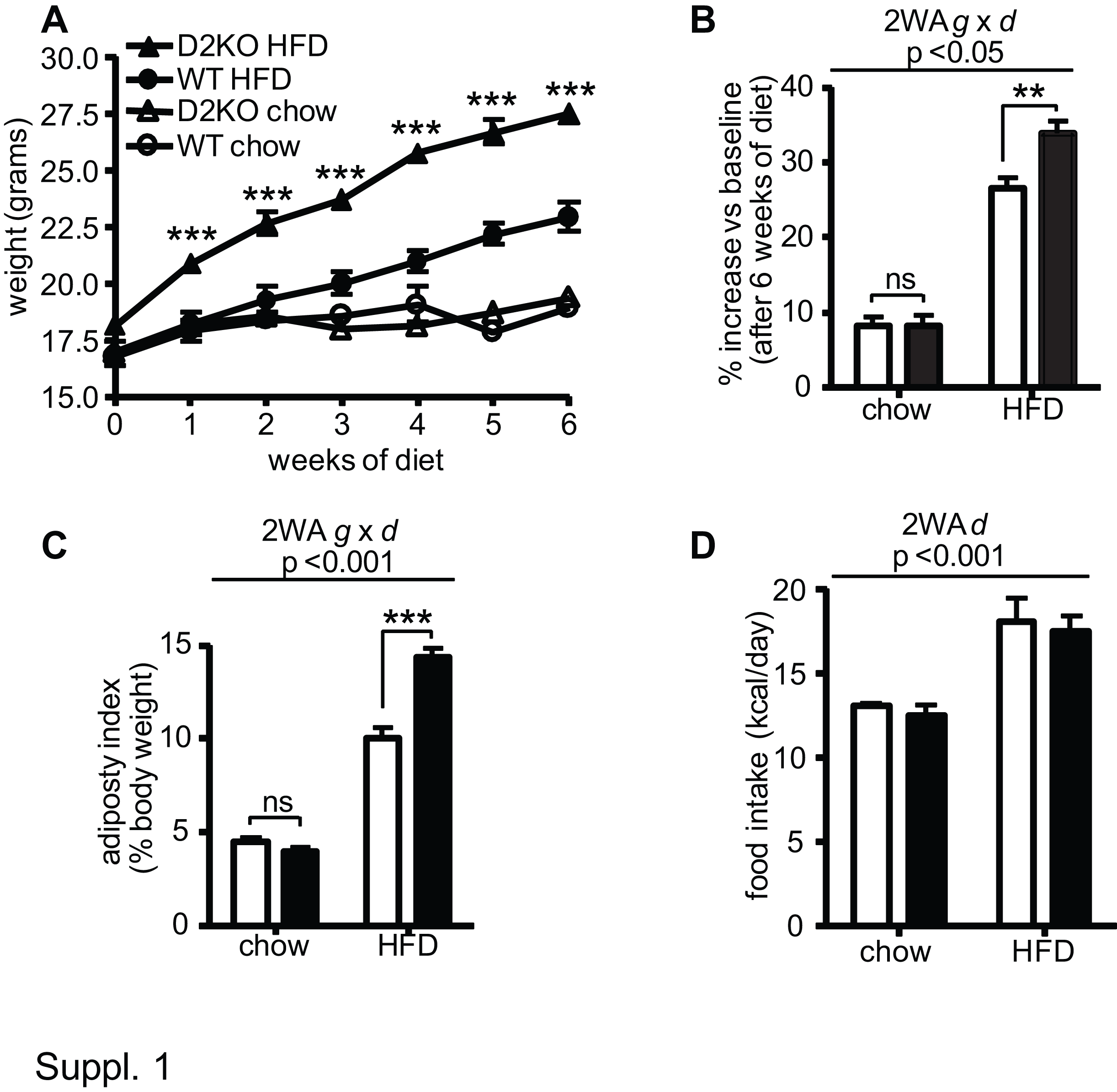

Supplement: Figure S1 — Female D2KO mice weight gain and body fat on a HFD. (A) Body weight of wild type and D2KO mice on chow or a HFD for 6-weeks. n = 4–5 mice/group. A significant interaction between genotype and diet was determined by two-way ANOVA for repeated measures (p<0.001; F = 21.02; Df = 96). After Bonferroni correction, there was a significant difference in weight gain of D2KO vs. WT starting at week 2 (B) Weight gain expressed at % of initial weight after 6 weeks on either chow or a HFD of mice shown in (A), two-way ANOVA indicated a significant interaction between genotype and diet (2WA g×d) (p<0.05; F = 7.52; Df = 16). (C) Weight of dissected perigonadal, mesenteric, perirenal, subcutaneous and brown adipose tissue fat depots divided by total body weight was used to calculate adiposity index as in [18], two-way ANOVA indicated a significant interaction between genotype and diet (2WA g×d) (p<0.0001; F = 37.52; Df = 16). (D) Average food intake of wild type and D2KO mice on either a chow or HFD was monitored for 7 days at 3 weeks of diet and is shown expressed as kcal/day/mouse. Two-way ANOVA indicated diet significantly effected the overall caloric consumption independent of genotype (2WA d) (p<0.001; F = 15.41; Df = 16). Data shown are the mean ± SEM, * = p<0.05, ** = p<0.01, *** = p<0.001, ns = not significant. (TIF) [file pone.0020832.s001.tif]

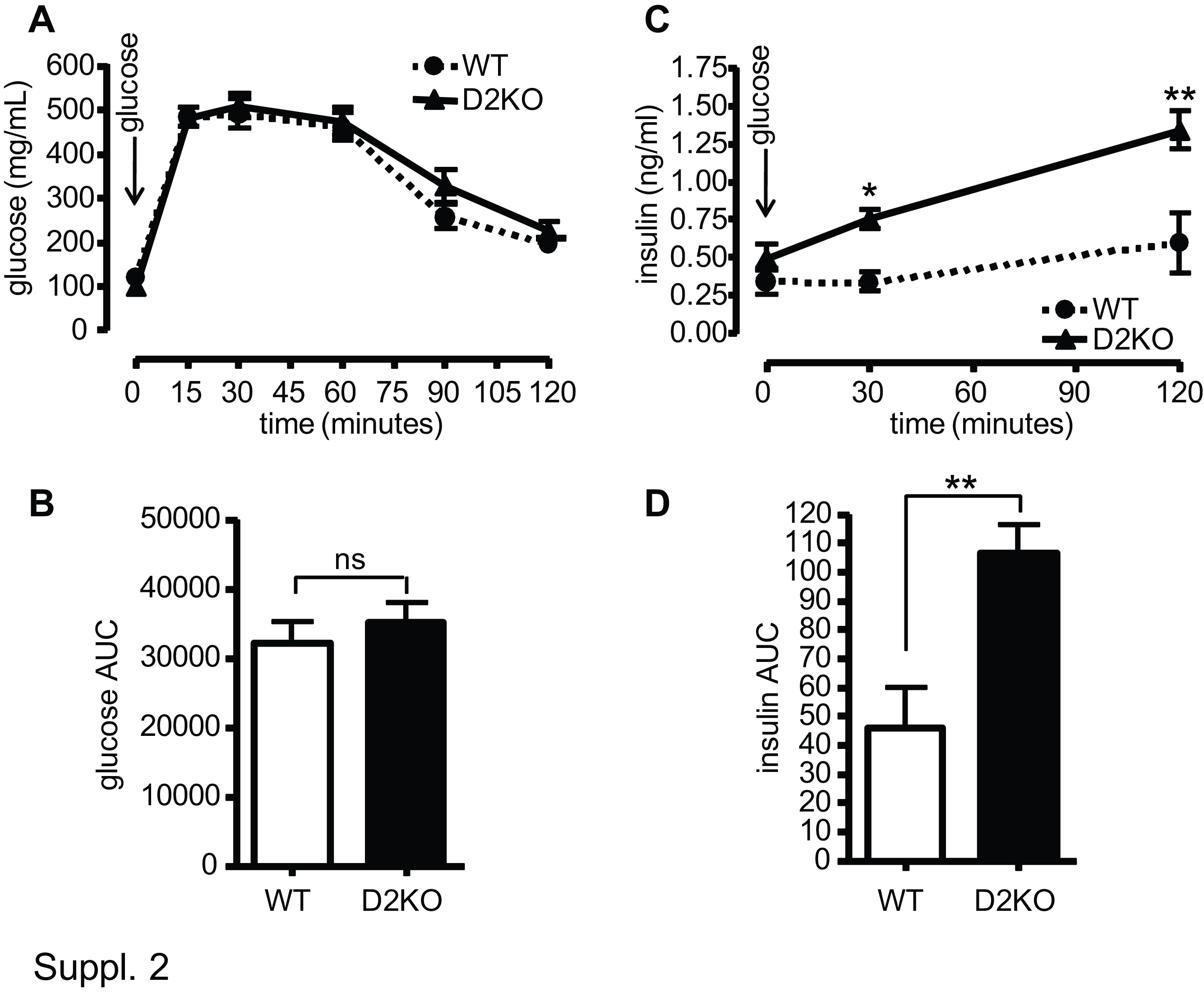

Supplement: Figure S2 — Glucose tolerance testing and corresponding insulin levels of female wild type and D2KO mice on a HFD. Female wild type and D2KO mice were maintained for 6-weeks on a HFD prior to testing. (A) Results of glucose tolerance testing performed as in Fig. 3A are shown. Blood glucose levels were measured at the indicated time, and are not significantly different by two-way ANOVA for repeated measures. (B) Histograms representative of the area under the curve of (A). (C) Corresponding serum insulin levels of mice in (A) Two-way ANOVA for repeated measures showed a significant effect (p value<0.01; F = 8.15, Df = 16). After Bonferroni correction significant differences were found at 90′ and 120′ (p<0.05 and p<0.001) (D) Histograms representative of the area under the curve of (C). N = 4–5 mice/group, female mice were 15 weeks old time of at testing. Data shown are the mean ± SEM with * = p<0.05, ** = p<0.01; ns = not significant. (TIF) [file pone.0020832.s002.tif]
