# Supplementary material for: Mice with a Targeted Deletion of the Type 2 Deiodinase Are Insulin Resistant and Susceptible to Diet Induced Obesity
Source: PLoS One. 2011 Jun 16;6(6):e20832. doi: 10.1371/journal.pone.0020832 (PMC3116839; doi:10.1371/journal.pone.0020832)
Supplement: Table S1 — (DOC) [file pone.0020832.s003.doc]

Supplementary table 1. List of primers used in the study.

| PRIMER | SEQUENCE |
| --- | --- |
| αGPD FW | GTGTGCGATACCTCCAGAAG |
| αGPD REV | GTTGTGTTGTCCGTCATAGTAG |
| ACC1 FW | TCTGATTTGGGGATCTCTGG |
| ACC1 REV | TTCTGATCCCTTTCCCTCCT |
| ACC2 FW | AGAGGCTACATCGCCTACGA |
| ACC2 REV | CTCTCTCTTGGGCAACAAGG |
| CPT1α FW | AAACCCACCAGGCTACAGTG |
| CPT1α REV | TCCTTGTAATGTGCGAGCTG |
| CPT1β 1 FW | GTCGCTTCTTCAAGGTCTGG |
| CPT1β 1 REV | AAGAAAGCAGCACGTTCGAT |
| cyclophilin A FW | CGCCACTGTCGCTTTTCG |
| cyclophilin A REV | ACTTTGTCTGCAAACAGCTC |
| D2 FW | CTTCCTCCTAGATGCCTACAAAC |
| D2 REV | GGCATAATTGTTACCTGATTCAGG |
| GLUT2 FW | GCCTGTGTATGCAACCATTG |
| GLUT2 REV | TCTCTGAAGACGCCAGGAAT |
| GLUT4 FW | TTGGAGAGAGAGCGTCCAAT |
| GLUT4 REV | CTCAAAGAAGGCCACAAAGC |
| Gys FW | AAGAGAAGCAACTCGGTGGA |
| Gys REV | AGATGTGTATCACCGCACCA |
| HK FW | AGAACCGTGGACTGGACAAC |
| HK REV | CAGACACTTGAAGGGGGTGT |
| HSL FW | TGAGATGCCACTCACCTCTG |
| HSL REV | GCCTAGTGCCTTCTGGTCTG |
| LPL FW | TTTTCTGGGACTGAGGATGG |
| LPL REV | TGGGAGCAAATGATTCCTTC |
| MCD | GGCCACTAATGAGGCTGTGT |
| MCD | TACCAGGCTGAGGATCTGCT |
| PDK2 FW | CTGGACCGCTTCTACCTCAG |
| PDK2 REV | GTTGGTGGCATTGACTTCCT |
| PDK4 FW | CCTTTGGCTGGTTTTGGTTA |
| PDK4 REV | CCTGCTTGGGATACACCAGT |
| PEPCK FW | CTCAACTCCCTCTGGCTTTG |
| PEPCK REV | ATACTGGCCACCACGTAAGC |
| PGC1α FW | ACAGCTTTCTGGGTGGATTGAAGTGG |
| PGC1α REV | AGACTGTCCAGTGTCTCTGTGAGGA |
| PPARα FW | CCAACATGGTGGACACAGAG |
| PPARα REV | GGCCTTGACCTTGTTCATGT |
| PPARγ FW | AGATTCTCCTGTTGACCCAGAGCA |
| PPARγ REV | AGGGCTTGATGTCAAAGGAATGCG |
| SERCA2 FW | TGGAGAACGCTCACACAAAG |
| SERCA2 REV | CACCACCACTCCCATAGCTT |
| UCP1 FW | ACAGAGCTGGTAACATATGACCTC |
| UCP1 REV | CCGGCAACAAGAGCTGACAGTAAA |
| UCP3 FW | AGAAGTTGCTGGAGTCTCACCTGT |
| UCP3 REV | GGAGCGTTCATGTATCGGGTCTTT |
